# Establishing the feasibility of the dosimetric compliance criteria of RTOG 1308: phase III randomized trial comparing overall survival after photon versus proton radiochemotherapy for inoperable stage II-IIIB NSCLC

**DOI:** 10.1186/s13014-016-0640-8

**Published:** 2016-05-04

**Authors:** Tawfik Giaddui, Wenzhou Chen, Jialu Yu, Liyong Lin, Charles B. Simone, Lulin Yuan, Yutao U. T. Gong, Q. Jackie Wu, Radhe Mohan, Xiaodong Zhang, Jaques B. Bluett, Michael Gillin, Kevin Moore, Elizabeth O’Meara, Jennifer Presley, Jeffrey D. Bradley, Zhongxing Liao, James Galvin, Ying Xiao

**Affiliations:** Sidney Kimmel Medical College, Thomas Jefferson University, Philadelphia, PA USA; University of Pennsylvania, Philadelphia, PA USA; Duke University, Durham, NC USA; MD Anderson Cancer Center, Houston, TX USA; University of California, San Diego, CA USA; Imaging and Radiation Oncology Core (IROC) Philadelphia-RT QA Center, Philadelphia, PA USA; Washington University School of Medicine, St. Louis, MO USA

**Keywords:** RTOG 1308, Dosimetric criteria, IMRT, Protons, PSPT

## Abstract

**Background:**

To establish the feasibility of the dosimetric compliance criteria of the RTOG 1308 trial through testing against Intensity Modulation Radiation Therapy (IMRT) and Passive Scattering Proton Therapy (PSPT) plans.

**Methods:**

Twenty-six lung IMRT and 26 proton PSPT plans were included in the study. Dose Volume Histograms (DVHs) for targets and normal structures were analyzed. The quality of IMRT plans was assessed using a knowledge-based engineering tool.

**Results:**

Most of the RTOG 1308 dosimetric criteria were achieved. The deviation unacceptable rates were less than 10 % for most criteria; however, a deviation unacceptable rate of more than 20 % was computed for the planning target volume minimum dose compliance criterion. Dose parameters for the target volume were very close for the IMRT and PSPT plans. However, the PSPT plans led to lower dose values for normal structures. The dose parameters in which PSPT plans resulted in lower values than IMRT plans were: lung V_5Gy_ (%) (34.4 in PSPT and 47.2 in IMRT); maximum spinal cord dose (31.7 Gy in PSPT and 43.5 Gy in IMRT); heart V_5Gy_ (%) (19 in PSPT and 47 in IMRT); heart V_30Gy_ (%) (11 in PSPT and 19 in IMRT); heart V_45Gy_ (%) (7.8 in PSPT and 12.1 in IMRT); heart V_50%_ (Gy) (7.1 in PSPT and 9.8 in IMRT) and mean heart dose (7.7 Gy in PSPT and 14.9 Gy in IMRT).

**Conclusions:**

The revised RTOG 1308 dosimetric compliance criteria are feasible and achievable.

## Introduction

Lung cancer is the main cause of cancer death in the United States (US) [1, 2]. In the year 2015, a total of 221,200 new cases and 158,040 deaths from lung cancer are estimated in the US [3]. Non-small cell lung cancer (NSCLC) accounts for about 80–85 % of these cases [1–3] and approximately 30 % of them are considered to be locally advanced, comprising both stage IIIA and IIIB in the current American Joint Committee on Cancer (AJCC) staging system [1]. Radiation therapy plus chemotherapy is the accepted standard of care for patients with locally advanced NSCLC. Proton therapy generally allows for reduced doses to organs at risk compared with photon therapy due to the physical properties of the proton beam, with essentially no dose delivered distal to the characteristic Bragg Peak [4, 5]. RTOG 1308 is a phase III randomized trial comparing overall survival after photon versus proton chemoradiotherapy for inoperable stage II-IIIB NSCLC. The trial’s purpose is to determine if proton therapy can improve overall survival over IMRT by reducing the risk of severe toxicity to organs at risk as compared to photon therapy, and new and more stringent dose constraints were employed [2]. The compliance criteria of clinical trials are often used as constraints for treatment planning and are used for plan scoring upon final review. The method used for designing compliance criteria should optimally examine a large number of treatment plans that are considered to be the result of a significant effort on the part of experienced treatment planners. In this paper, we assessed the feasibility of the new and more stringent dosimetric criteria of the RTOG 1308 trial using the IMRT and PSPT plans submitted during the design stage of the trial. This study aims at improving the efficiency of clinical trials launch by establishing realistic dose constraints in advance.

## Methods and materials

### Dosimetric compliance criteria for RTOG 1308 trial

The total prescribed dose will be up to 70 Gy (RBE) without exceeding the tolerance dose-volume limits of all critical normal structures. The compliance criteria used for two earlier RTOG trials—RTOG 0617 [1] and RTOG 1106 [6]—were used to develop the compliance criteria for the RTOG 1308 trial. In fact, RTOG 0617 findings indicated that overall survival was associated with doses to organs at risk (OARs) [7]. RTOG 1308 dosimetric compliance criteria were, therefore, designed with a plan to enforce more stringent dose constraints. Additionally, QUANTEC recommendations [8–11] were considered when dose volume constraints for normal critical structures were developed. Table 1 lists the RTOG 1308 dosimetric constraints.

**Table 1:** RTOG 1308 dosimetric compliance criteria for target volumes and normal structures

RT Parameter	Per protocol	Variation acceptable
% of PTV covered by prescription dose	95 %	≥95 % of the PTV is covered by ≥95 % of the prescription dose
% of ITV (motion-incorporated CTV)	100 %	≥99 %
Maximum PTV dose PTV (0.03 cc)	≤120 % RX	≤125 % RX
Minimum PTV dose (0.03 cc)	≥85 % RX	≥75 % RX
Normal lung (Both lungs minus GTV)	V5 ≤ 60 %	V5 ≤ 65 %
	V20 ≤ 37 %	V20 ≤ 40 %
	Mean dose < 20 Gy (RBE)	Mean Dose: ≤ 22 Gy (RBE)
Esophagus	Max dose: 74 Gy (RBE) ≤ 1 cc of partial circumference	Max dose: 74 Gy (RBE) ≤ 1.5 cc of partial circumference
Brachial Plexus	V66 ≤ 2.0 cc	V66 ≤ 2.5 cc
	V70 ≤ 1.0 cc	V70 ≤ 1.5 cc
	V74 ≤ 0.5 cc	V74 ≤ 1.0 cc
	V75 ≤ 0.1 cc	V75 ≤ 0.5 cc
Spinal Cord	V50 < 0.03 cc	V52 < 0.03 cc
Heart	V30 ≤ 50 %	50 % ≤ V30 ≤ 55 %
	V45 ≤ 35 %	35 % ≤ V45 ≤ 40 %

*RX* prescription dose

### Target definitions and treatment planning

The study investigated 26 patients, each of whom had an IMRT plan and PSPT plan and plans were chosen for consecutive patients. Clinical proton and photon plans were generated for each patient either with the intention of delivering the more optimal plan based on DVH parameters or as comparison plans for insurance purposes. The plans were submitted by two institutions, with the first institution (The University of Pennsylvania) submitting plans for 16 consecutive patients, and the second institution (MD Anderson Cancer Center) submitting plans for 10 consecutive patients. The contouring of normal structures and target volumes as well as motion management was performed in accordance with guidelines from RTOG 1308 at both institutions.

The gross tumor volume (GTV) (for both the primary tumor and nodal metastases) was contoured based on findings from the computed tomography (CT), positron emission tomography (PET)/CT scan, and pathology. An iGTV was created to account for the respiratory motion of the GTV using an eight-phase four-dimensional (4D) simulation scan by the first institution and a 10-phase 4D CT simulation scan by the second institution. An internal target volume (ITV) was then created by expanding the iGTV by 3–5 mm for sites of nodal metastases and 8 mm for the primary tumor without extending into uninvolved organs (such as esophagus, heart, or bone). A planning target volume (PTV) was created by expanding the ITV isotropically by 5 mm. The same ITVs and PTVs, as well as the same contours for normal structures, were used by PSPT and IMRT plans for each patient. Table 2 lists the mean and range of volumes in cubic centimeters (cc) for all structures used in this study. The energy was 6 MV in all IMRT plans. For proton plans, beam range compensators were developed to account for range uncertainties, and they provided proximal and distal margins relative to each PTV. Each PTV had a unique blocking developed to create a lateral margin relative. Two to four fields were used for each proton plan, with beam angles and energies dependent on the target volume location and size. Most of the 16 patients had treatment plans (planned by the first institution) that were coplanar, except for one of the proton plans and three IMRT plans. The block margin of the proton multileaf collimators (MLC) was comparable with beam penumbra. The first institution used the Varian™ Eclipse treatment planning system (Varian Medical Systems, Palo Alto, CA) (dose volume optimizer [11.0.30], anisotropic analytical algorithm for dose calculation [AAA; 11.0.30] for IMRT plans, and proton convolution superposition [PCS; 10.0.28] for proton plans). The second institution used various versions of Philips Pinnacle (Philips Health Care) to generate photon plans as well as Variance Eclipse™ (V. 8.9) to generate proton plans.

**Table 2:** Volumes in cubic centimeters (cc) for all structures (target volume and normal structures) used in this study

Structure	Minimum	Maximum	Median	Mean ± SE
Target volume	76.55	1160.83	420.5	548 ± 60
Normal Lung	1328.66	5701.44	2860.8	3033 ± 183
Heart	396.54	1084.13	676.96	671 ± 37
Esophagus	21.59	66.97	33.73	35 ± 2
Spinal Cord	14.25	71.59	37.95	39 ± 3

*SE* standard error

### Quality assurance of treatment plans using knowledge-based engineering tool

The prescription dose was 66.6/1.8 Gy for plans that the first institution submitted and 74 Gy for plans that the second institution submitted. As such, all plans were scaled so that a prescription dose of 70/2 Gy RBE covered 95 % of the PTV (as per RTOG 1308 guidelines). The scaling factor for plans that the first institution provided ranged from between 1.06 and 1.12, and the scaling factor for plans that the second institution submitted ranged from between 0.93 and 1.00. The quality of all scaled plans was evaluated using a knowledge-based engineering (KBE) tool [12–14]. The tool generates dose volume histogram for OARs based on the patients’ anatomical information using predictive models. The predictive models correlates the anatomical features with the dose features embedded in the IMRT plans and was trained using a multi-institutional database of past high-quality plans. The OARs and their anatomical features were analyzed upon building the model and a stepwise multivariate regression was used to assess the significance of the features and establish the models that correlate anatomy features and dosimetry features. The details of the model building and evaluation is described in [13]. The model was later validated by comparing the DVHs predicted by the model with the actual DVHs of clinical plans for the following dosimetric parameters, V5Gy, V10Gy, V20Gy of Lung, mean dose of heart and esophagus. DICOM data (CT, RtPlan, RtDose and RtStruc) of all IMRT plans used in this study were imported to the KBE tool. The tool requires the matching of PTV and OARs names in the candidate plan with the names used in the models. The tool then generates a modeled dose volume histogram (DVH) for organs at risks for the evaluation plan in study. The predicted DVHs were compared with the planned DVHs to assess the quality of plans involved [15].

### Data analysis

DVHs for all plans were analyzed using MIM Software, Inc. [16]. Dose parameters from the two types of plans were compared using a paired t-test. Differences were considered significant if *p* < 0.05 (two-sided).

## Results

### Quality assurance of photon IMRT plans using knowledge-based engineering tool

Table 3 lists the various dosimetric parameters of different OARs, as calculated by the KBE tool and treatment planning system. The dosimetric parameters for all OARs, as calculated by the plans, are either less than those predicted by the tool or within the confidence limits of the predicted values. This indicates that the treatment plans used in this study are of good quality; also, the scaling of plans to the prescription dose of RTOG 1308 did not affect their quality.

**Table 3:** Comparison of OARs dosimetric criteria as estimated by the treatment planning system and the knowledge based engineering tool

Dosimetric Criteria	Treatment planning system	Knowledge based engineering tool
Spinal Cord Maximum Dose (Gy)	40 ± 2	44 ± 2
Esophagus Maximum Dose (Gy)	68 ± 3	75 ± 2
Heart V_30 Gy_ (%)	16 ± 4	15 ± 3
Heart V_45 Gy_ (%)	10 ± 3	9 ± 2
Lung V_20Gy_ (%)	27 ± 2	29 ± 2
Lung mean dose (Gy)	17 ± 1	18 ± 2

### Compliance of IMRT and PSPT plans to RTOG 1308 dosimetric criteria

Table 4 lists the percentage deviation unacceptable rates (i.e., failed to comply with the protocol’s dosimetric criteria) for the different dosimetric criteria of RTOG 1308. The deviation unacceptable rates for PTV minimum dose (will be referred to as PTVmin in the rest of the manuscript) were 23 and 27 % for IMRT and PSPT plans, respectively. All IMRT plans met the PTV maximum dose (will be referred to as PTVmax in the rest of the manuscript) criteria, and only 4 % of PSPT plans failed to meet these criteria (scored deviation unacceptable). The deviation unacceptable rates in lung V_5Gy_ (%) and V_20Gy_ (%) compliance criteria were 4 %, and in the mean lung dose (will be referred to as MLD in the rest of the manuscript), the rate was 12 % for IMRT plans. All PSPT plans met the lung V_5Gy_ (%) and V_20Gy_ (%) compliance criteria, and only 4 % failed to meet the MLD criteria (scored deviation unacceptable). The deviation unacceptable rates in the heart V_30Gy_ (%) and V_45Gy_ (%) were 8 and 4 %, respectively, in IMRT plans; meanwhile, all PSPT plans met the heart V_30Gy_ (%) and V_45Gy_ (%) compliance criteria. One IMRT plan failed to meet the spinal cord maximum dose criteria, but this constraint was met in all PSPT plans.

**Table 4:** Deviation unacceptable rates (expressed as percentage) for the different dosimetric criteria

Structure	Dosimetric Parameter	Photon IMRT	Proton PSPT
		% of cases failed to meet the criteria	% of cases failed to meet the criteria
PTV	% of PTV covered by RX	0	0
	PTV minimum	23	28
	PTV maximum	0	4
Spinal Cord	Maximum dose	4	0
Total Lung (both lungs – GTV)	V5	4	0
	V20	4	0
	Mean dose	12	4
Heart	V30	8	0
	V45	4	0
Brachial Plexus	V70	10	5
	V74	10	0
	V75	5	0

### Photon IMRT and proton PSPT dose volume histogram analysis

**Fig. 1:**
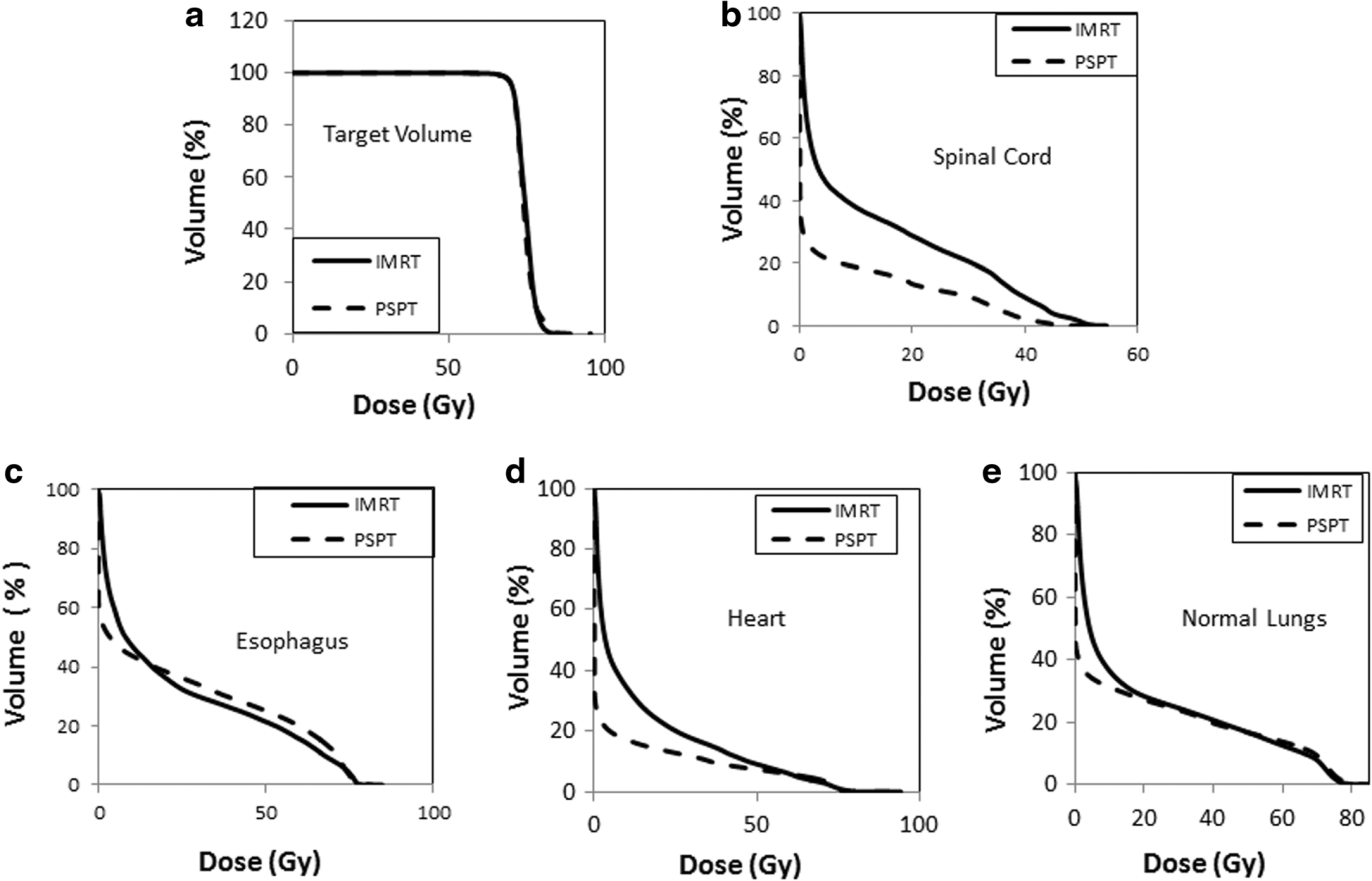
Average dose volume histograms of IMRT and PSPT plans. **a** Target Volume. **b** Spinal Cord. **c** Esophagus. **d** Heart. **e** Normal Lung

Figure 1 shows the average dose volume histograms for the target volume, spinal cord, heart, esophagus, and normal lung in IMRT and PSPT plans (the average dose volume histogram of each plan type is calculated using the DVHs of all cases; it represents the arithmetic mean of the percentage volume at every dose point). Table 5 summarizes the DVH analysis of IMRT and PSPT plans. A general and observable feature in all PSPT DVHs of normal structures (Fig. 1) was the steep initial drop in the percentage volume receiving a certain dose, as compared with IMRT DVHs. The spinal cord percentage volumes receiving a given dose were around 65 % less in PSPT than in IMRT plans over almost the entire dose range. For the heart, the percentage volume receiving a given dose was about 35 % less in the PSPT plans than in the IMRT plans up to about 60 Gy. It then became 18 % higher than that of IMRT plans over the rest of the dose range (from about 60 Gy up to about 80 Gy). The esophagus percentage volume receiving a given dose was 14 % higher in IMRT plans than in PSPT plans up to about 15 Gy; it then became 19 % higher in PSPT plans up to almost 75 Gy. The normal lung percentage volumes receiving a given dose was higher in IMRT plans than in PSPT plans up to about 20 Gy; they then became comparable over a dose range from 20 Gy up to about 55 Gy, when it became slightly higher in PSPT plans. It is observed that the values of lung V_5Gy_ (%), lung V_20Gy_ (%), MLD, spinal cord maximum dose, heart V_5Gy_ (%), heart V_30Gy_ (%), heart V_45Gy_ (%), and heart mean dose were lower in the PSPT plans when compared with IMRT plans. Lung V_5Gy_ (%) and MLD were significantly lower (27 % [*p* < 0.001] and 7.2 % [*p* < 0.001], respectively) in the PSPT plans. The lung V_20Gy_ (%) was 1.6 % (*p* = 0.189) less in PSPT plans. The mean spinal cord maximum dose was significantly less (27 % [*p* < 0.001]) in PSPT plans than in IMRT plans. Heart V_5Gy_ (%), mean heart dose, V_30Gy_ (%), V_45Gy_ (%), and V_50Gy_ (%) were 59 % (*p* < 0.001); 48 % (*p* < 0.001); 41 % (*p* <0.001); 35 % (*p* = 0.029); and 27 % less in PSPT plans when compared with IMRT plans. On the contrary, heart V_60Gy_ (%), V_66Gy_ (%), and V_70Gy_ (%) were, respectively, 8; 13, and 18 % higher in PSPT plans as compared with IMRT plans. The mean values for the esophagus mean and maximum doses were very close (1–2.5 %) in both the IMRT and PSPT plans. The inferior performance of high dose in heart, lung and esophagus in PSPT is due to the limitation of PSPT in changing field portal at different proton energies [17].

**Table 5:** Analysis of dose volume histograms of IMRT and PSPT plans

Structure		Photon IMRT		Proton PSPT	
		Median	Mean ± SE	Median	Mean ± SE
PTV	% of PTV covered by RX	95	95	95	95
	PTV minimum	57.0	56.7 ± 1.5	55.0	55.5 ± 1.4
	PTV maximum	81.3	81.6. ± 0.7	79.9	80.9 ± 0.9
Normal lung (total lungs – GTV)	Lung V5	42.6	47.2 ± 2.6	35.3	34.4. ± 1.6
	Lung V20	27.9	28.1 ± 1.6	29.32	27.6 ± 1.5
	Mean Lung dose	18.4	17.8 ± 0.9.	18.3	16.4. ± 0.9.
Spinal cord	Spinal Cord maximum dose	43.8	43.5 ± 1.4	37.9	31.7 ± 3.1
Heart	V5	39.4	47 ± 6	16.6	19. ± 3
	V30	15.7	19 ± 4	9.7	11 ± 2
	V45	7.7	12.1 ± 2.7	7.4	7.8 ± 1.5
	V50	5.4	9.8. ± 2.3	6.5.	7.1 ± 1.4
	V60	2.6	6.2 ± 1.5	4.2	6.7 ± 1.2
	V66	1.8	4.2 ± 1	2.9	4.7 ± 1.1
	Heart mean dose	12.7	14.9 ± 2.4	6.7	7.7. ±1.4
	Maximum Dose	78	69.3. ± 4.5	76.4	68.5. ± 4.4.
Esophagus	Mean dose	20.1	22.7 ± 1.9	21.2	23 ± 2
	Maximum dose	76.2	73.2 ± 2.5	74.1	71. ± 3
Brachial Plexus	V70	0	0.50	0	1.0
	V74	0	0.27	0	0.10
	V75	0	0.18	0	0.11

*SE* standard error

## Discussion

### Quality assurance of treatment plans using knowledge-based engineering tool

The knowledge-based engineering tool is a quick and reliable tool for checking the quality of treatment plans. This tool [12] was trained using a multi-institutional dataset of high-quality plans to take into consideration the variations in the anatomies of patients, institutional protocols, and treatment techniques. Treatment plans are considered to be of good quality if their calculated dosimetric parameters are less than the values predicted by the tool or if they lie within the confidence level (uncertainty level) of the predicted ones. The quality of all IMRT plans was assessed using this tool. As indicated in Table 4, the values of various dosimetric parameters (volumes receiving a certain dose; mean dose and maximum dose of various structures) as calculated by the treatment plans were within the uncertainty of the values predicted by the tool, indicating that the quality of all plans are generally acceptable. This is a very important step for ensuring that the plans used for establishing the dosimetric criteria are not only acceptable but also of good quality, and it also indicates that the scaling of the plans to the prescription dose of RTOG 1308 did not affect their quality.

### Compliance of IMRT and PSPT plans to RTOG 1308 dosimetric criteria

This study was conducted when designing the new and more strict dose constraints now in place for the RTOG 1308 clinical trial to test if these criteria are achievable; based on its findings, many criterion were relaxed (for example, the minimum dose of PTV) or removed (for example, the maximum heart dose of 74 Gy; this was removed after protocol enrollment already started based on the current study findings). Such a study represents a practical improvement in the trial planning processes. By establishing in advance that dosimetric compliance criteria are achievable across a variety of centers and techniques. RTOG and now NRG Oncology clinical trials usually specify two levels of constraints: per protocol and variation acceptable. Ideally, all plans are expected to meet the per-protocol constraints; however, plans are still considered acceptable if there is a minor deviation from the per-protocol constraints and it is still within the range of the variation-acceptable constraints. As a general rule, if the deviation unacceptable rates significantly exceed 10 %, the criterion has to be revised. The deviation unacceptable rates for all RTOG 1308 criteria were less than 10 %, with the exception of the PTVmin dose and mean lung dose (MLD). We compared the PTVmin of the plans with that required by the protocol (Table 2) and noticed that the deviations of the plans from the protocol were less than 5 % in four IMRT plans and ranged between 20 and 34 % in the other two cases. In the PSPT plans, the deviation ranged from between 2.9 and 4.7 % in two cases, ranged from between 8 and 12 % in four cases, and reached 20 % in one case. This effect is likely due to lack of a PTVmin institutional constraint at the time the plans were developed. The deviations of the IMRT plans that failed the MLD criterion from the protocol dose constraints ranged from between 11 and 20 %. Only one PSPT plan failed to meet the MLD criterion, and the deviation of the plan was 15 % from the protocol dose constraints. Such deviation unacceptable rates in some of the plans were dealt with using the protocol allowable variations (prescribing 95 % of the prescription dose to 95 % of PTV or using a prescription dose of 60 Gy instead of 70 Gy) [2].

### Photon IMRT and proton PSPT dose volume histogram analysis

The trend of our results agreed qualitatively with those reported by Berman et al.[18] for lung V_5Gy_ (%), mean lung dose, spinal cord maximum dose, and esophagus mean dose. However, a disagreement was observed between our results and Berman et al.[18] on the mean heart dose and the lung V_20Gy_ (%): They reported 4.6 % higher mean heart dose in PSPT plans as compared with IMRT plans, whilst in our study, the mean heart dose was 48 % less in PSPT plans as compared with IMRT plans. The lung V_20Gy_ (%) was 2.7 % higher in PSPT plans compared with in IMRT plans in Berman et al.’s [18], while it was 1.6 % higher in IMRT plans as compared with PSPT plans in our investigation. Chang et al. [19] compared photon and proton plans for stage III NSCLC patients and reported a reduction of 9–3 Gy (compared with 12 Gy in our investigation) in the spinal cord maximum dose and 3 Gy (compared with 1.31 Gy in our study) in the mean lung dose when proton beams were used as compared with IMRT. They also reported 15-17 % (12 % in our study) and 4 % (~0.5 % in our study) in lung V_5Gy_ (%) and lung V_20Gy_ (%), respectively. The prescription dose was different in Chang et al.’s [19] study; they used two different dose levels: 60–63 Gy (RBE) and 74 Gy (RBE) for protons and photons, respectively. Kesarwala et al. [20] examined the feasibility of intensity-modulated proton therapy (IMPT) for elective nodal irradiation in locally-advanced none small-cell lung cancer. They reported a mean lung dose of 17.2 ± 0.9 (Gy/CGE) (compared to 17.8 ± 0.9 Gy in our IMRT results) using photon IFRT and 11 ± 0.8 Gy/CGE (compared to 16.4 ± 0.9 Gy in our PSPT results) using IMPT; our results on IMRT are comparable to their results. They also reported lung V_20Gy_ of 27.9 ± 1.6 % (compared to 28.1 ± 1.6 % in our IMRT plans) and 22.9 ± 1.5 % (compared to 27.6 ± 1.5 % in our PSPT plans). Our IMRT plans results are in good agreement with their results. However, there are some differences in the proton plans, as they used IMPT, which resulted in lower mean lung dose and lung V20Gy.

## Conclusion

Most of the dosimetric criteria were achieved using the IMRT and PSPT plans, suggesting that the RTOG 1308 dosimetric compliance criteria are feasible and achievable despite the relatively high deviation unacceptable rate in the PTVmin dose compliance. The KBE QA tool indicated that the plans used in this study are of good quality. PSPT plans led to a significantly lower heart V_5Gy_ (%), V_30Gy_ (%), V_45Gy_ (%), V_50Gy_ (%), heart mean dose, lung V_5Gy_ (%), spinal cord maximum dose, and esophagus maximum dose as compared with IMRT plans. PSPT led to a numerically slightly higher heart V_60Gy_ (%), V_66Gy_ (%), brachial plexus V_70Gy_ (CC), and esophagus mean dose; however, these differences were within statistical uncertainty.
